# Biomimetic Anti‐PD‐1 Peptide‐Loaded 2D FePSe_3_ Nanosheets for Efficient Photothermal and Enhanced Immune Therapy with Multimodal MR/PA/Thermal Imaging

**DOI:** 10.1002/advs.202003041

**Published:** 2020-11-25

**Authors:** Xueyang Fang, Xianlin Wu, Zhendong Li, Lijun Jiang, Wai‐Sum Lo, Guanmao Chen, Yanjuan Gu, Wing‐Tak Wong

**Affiliations:** ^1^ Department of Applied Biology and Chemical Technology The Hong Kong Polytechnic University Hong Kong SAR P. R. China; ^2^ Institute of Clinical Medicine The First Affiliated Hospital of Jinan University Guangzhou 510632 P. R. China; ^3^ Pancreatic Disease Diagnosis and Treatment Center Jinan University Guangzhou 510632 P. R. China; ^4^ Hepatobiliary Surgery Department The First Affiliated Hospital of Jinan University Guangzhou 510632 P. R. China; ^5^ Medical Imaging Center First Affiliated Hospital of Jinan University Guangzhou 510630 P. R. China

**Keywords:** cancer photothermal–immune therapy, FePSe_3_ nanosheets, metal phosphorus trichalcogenides, multimodal imaging, PD‐1 blockade

## Abstract

Metal phosphorous trichalcogenides (MPX_3_) are novel 2D nanomaterials that have recently been exploited as efficient photothermal–chemodynamic agents for cancer therapy. As a representative MPX_3_, FePSe_3_ has the potential to be developed as magnetic resonance imaging (MRI) and photoacoustic imaging (PAI) agents due to the composition of Fe and the previously revealed PA signal. Here, a FePSe_3_‐based theranostic agent, FePSe_3_@APP@CCM, loaded with anti‐PD‐1 peptide (APP) as the inner component and CT26 cancer cell membrane (CCM) as the outer shell is reported, which acts as a multifunctional agent for MR and PA imaging and photothermal and immunotherapy against cancer. FePSe_3_@APP@CCM induces highly efficient tumor ablation and suppresses tumor growth by photothermal therapy under near‐infrared laser excitation, which further activates immune responses. Moreover, APP blocks the PD‐1/PD‐L1 pathway to activate cytotoxic T cells, causing strong anticancer immunity. The combined therapy significantly prolongs the lifespan of experimental mice. The multimodal imaging and synergistic therapeutic effects of PTT and its triggered immune responses and APP‐related immunotherapy are clearly demonstrated by in vitro and in vivo experiments. This work demonstrates the potential of MPX_3_‐based biomaterials as novel theranostic agents.

## Graphical abstract

In this work, it is shown that the novel biomimetic nanosystem FePSe_3_@APP@CCM can be used for magnetic resonance/photoacoustic imaging‐guided synergistic anticancer photothermal therapy (PTT) and immunotherapy. The nanosheets (NSs) are decorated with cancer cell membranes and show immune escape and homologous targeting abilities. Under near‐infrared laser irradiation, these NSs provide direct PTT and multiple immunotherapies.

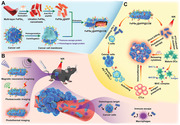

## 1 Introduction

Cancer immunotherapy by stimulating or mobilizing the body's inherent immune system to enhance anticancer effects has emerged as a new cancer treatment after surgical therapy, radiotherapy, and chemotherapy.^[^ ^1^, ^2^ ^]^ Immune checkpoint blockade, such as antiprogrammed cell death 1 (anti‐PD‐1) therapy, can block the immune evasion of cancer cells by inhibiting the activities of immunosuppressive T cells and reactivating tumor‐infiltrating cytotoxic T lymphocytes (CTLs) and has demonstrated clinical efficacy in several types of solid tumors.^[^ ^3^, ^4^, ^5^, ^6^ ^]^ Programmed cell death‐1 (PD‐1) protein is a type of immunosuppressive molecule highly expressed on activated T and B cells, while the corresponding receptor programmed cell death ligand‐1 (PD‐L1) is a transmembrane protein overexpressed on the surface of tumor cells. Blockage of the interaction between the PD‐1 protein and PD‐L1 has been reported to revoke T cell functions, leading to enhanced antitumor immunity. However, the efficacy of immunotherapy is often limited by a single immune activation, including efficient PD‐1/PD‐L1 immune checkpoint blockade. Therefore, immunotherapy containing multiple immune activations, such as photothermal therapy (PTT)‐ or photodynamic therapy (PDT)‐induced immunotherapy, would eradicate tumors more effectively through additional pathways. Additionally, the development of nanotechnology has been made possible for a single nanoplatform to achieve the improved cancer immunotherapy through immunostimulation and immunosuppression approaches.^[^ ^7^, ^8^ ^]^ More interestingly, nanotheranostics rationally designed by integrating immunotherapy with other therapeutic or imaging modalities into one system to achieve the goal of synergistic therapy and real‐time monitoring of treatment have gained more and more attentions.

PTT relies on photothermal transducing agents (PTAs) that absorb near‐infrared (NIR) light and convert it into heat to ablate tumors, which has been recognized as a promising cancer treatment modality.^[^ ^6^ ^]^ In addition to the direct ablation of tumor cells, PTT can trigger anticancer immune responses, which has been demonstrated as an approach to treat metastatic tumors by producing tumor‐associated antigens.^[^ ^9^, ^10^ ^]^ Therefore, it would be ideal to combine PTT and immunotherapy for cancer therapeutics.^[^ ^11^, ^12^, ^13^, ^14^ ^]^ Recently, inorganic PTAs coated with anti‐PD‐1/antiprogrammed cell death ligand 1 (anti‐PD‐L1) antibody or peptides have been successfully applied for combined cancer therapy.^[^ ^15^, ^16^, ^17^, ^18^, ^19^ ^]^

Recently, 2D nanomaterials have attracted widespread attention in theranostic biomedicine, including multimodal imaging, PTT, and PDT, due to their intriguing physiochemical properties, ultrathin structure, and large specific surface area. Different types of 2D nanomaterials, including graphene,^[^ ^20^, ^21^ ^]^ MXene,^[^ ^22^, ^23^, ^24^, ^25^ ^]^ black phosphorus,^[^ ^26^ ^]^ and transition metal dichalcogenides (MoS_2_, MoSe_2_),^[^ ^27^, ^28^ ^]^ have been developed as PTT agents for tumor ablation because of their high photothermal conversion efficiency, easy fabrication, and tunable optical properties. More recently, metal phosphorus trichalcogenides (MPX_3_) were identified as a new kind of 2D material with a general formula of MPX_3_ (where M is the transition metal such as Fe, Ni, Mn, Zn, Co, and Cd; X is S or Se) and have attracted increasing attention in various fields, such as catalysis, electronics, and magnetism.^[^ ^29^, ^30^, ^31^ ^]^ To the best of our knowledge, the first example of MPX_3_‐type molecule FePS_3_‐based nanosheets (NSs) being explored as anticancer agents was only reported very recently.^[^ ^32^ ^]^ These molecules were highly efficient in synergistic photothermal–chemodynamic therapy; however, the potency in magnetic resonance imaging (MRI) and photoacoustic imaging (PAI) remains unexplored. As a representative MPX_3_, FePSe_3_ has strong absorption in the NIR region, a wide bandgap, and a high photothermal conversion efficiency, allowing for cancer PTT.^[^ ^30^, ^32^ ^]^ Compared to the abovementioned 2D nanomaterials, the presence of Fe in 2D FePSe_3_ imparts MR imaging function without further integration of additional paramagnetic species such as iron or Mn. Therefore, the specific surface area of 2D FePSe_3_ could be loaded with immunotherapy‐associated molecules such as anti‐PD‐1 or anti‐PD‐L1 peptides and not other imaging components, thus improving the drug loading and therapeutic efficacy. Therefore, FePSe_3_ has the potential to become an “all‐in‐one” theranostic nanoplatform combining the possibilities of magnetic resonance imaging, photoacoustic imaging, and drug loading for multimodal diagnosis and synergistic therapy.

One main concern regarding the bioapplications of nanomaterials is the recognition by macrophages as foreigners, which results in rapid clearance by the reticuloendothelial system and severely limits nanomaterial targeting.^[^ ^33^, ^34^ ^]^ Cell membrane‐coating technology provides nanoplatforms with the ability to mimic source cells to alleviate immune recognition.^[^ ^35^, ^36^ ^]^ Several types of cell membranes have been reported for nanoparticle encapsulation,^[^ ^37^ ^]^ including stem cells,^[^ ^38^ ^]^ red blood cells,^[^ ^39^, ^40^ ^]^ leukocyte,^[^ ^41^, ^42^ ^]^ macrophage cells,^[^ ^43^, ^44^ ^]^ and cancer cells.^[^ ^45^, ^46^, ^47^, ^48^, ^49^ ^]^ Specifically, the cancer cell membrane (CCM) extracted from natural cancer cells can retain antigenic diversity for immune escape and the homotypic binding capacity that originates from the natural properties of cancer cells in facilitating adhesive interactions.^[^ ^50^, ^51^ ^]^ Therefore, CCM‐camouflaged nanomaterials could have homologous binding capability and immune escape ability at the same time and thus improve specific binding for targeting efficiency and decelerate immune clearance, providing an important strategy for effective diagnosis and therapy. For example, a recent study has employed CCM to coat immunoadjuvant‐loaded nanoparticles MANPs/R837 as a source of multiple antigens.^[^ ^49^ ^]^

**Scheme 1:**
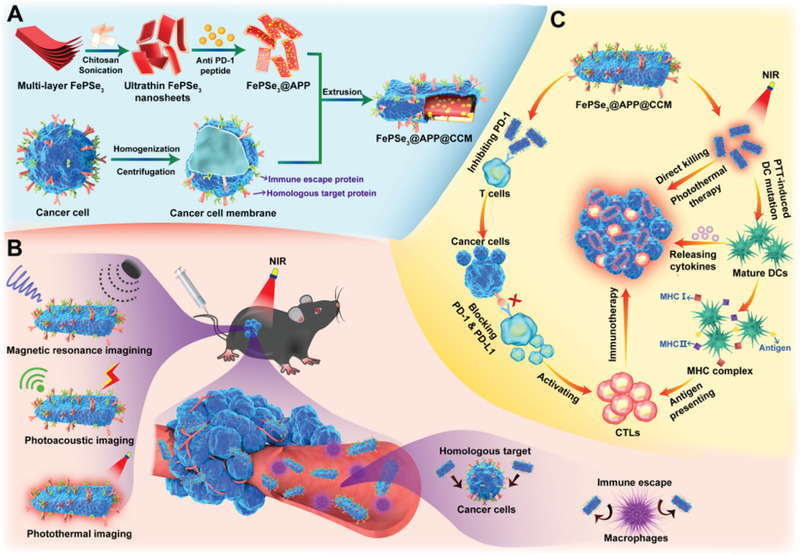
A) The design and preparation of FePSe_3_@APP@CCM NSs. B) FePSe_3_@APP@CCM with immune escape and homologous targeting abilities for effective tumor multimodal imaging. C) The mechanism of FePSe_3_@APP@CCM for synergistic cancer photothermal immunotherapy.

Herein, we report the first example of a biomimetic nanosystem constructed from FePSe_3_ for MR/PA imaging‐guided synergistic PTT‐immune combined therapy (**Scheme** **1**). The bulk FePSe_3_ was modified with chitosan (CS) for drug loading and improved stability. The anti‐PD‐1 peptide (APP) was then covalently bound to CS‐stabilized FePSe_3_ NSs to block the PD‐1/PD‐L1 pathway for immunotherapeutic effects. The CCM of CT26 cells was finally used to decorate the surface of FePSe_3_@APP NSs to provide the “all‐in‐one” NSs. Under NIR laser irradiation, the photothermal effects caused by FePSe_3_@APP@CCM not only directly killed cancer cells but also induced intense immune responses. Together with the peptide APP, this single 2D nanoplatform can induce multiple immune responses, thus promoting the release of cytokines or presentation of antigens to activate CTLs for efficient immunotherapeutic effects. Taken together, this study provides a feasible strategy for designing bioinspired 2D MPX_3_‐based NSs for multimodal imaging and synergistic cancer therapy.

## 2 Results and Discussion

### 2.1 Preparation and Characterization of FePSe_3_@APP@CCM NSs

**Figure 1:**
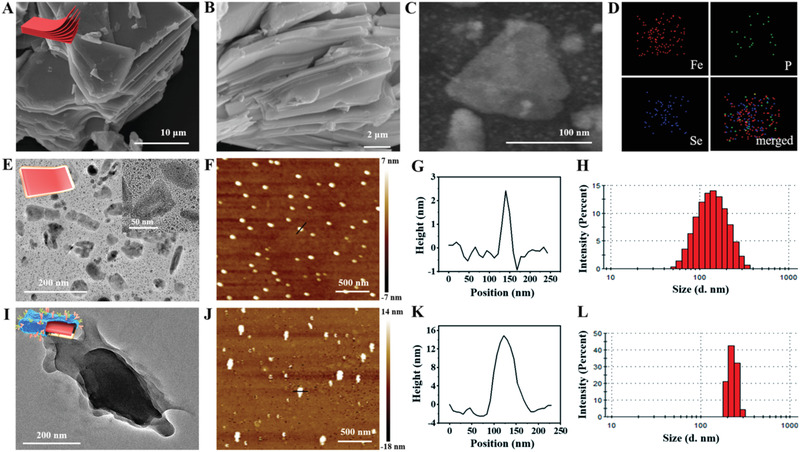
A,B) SEM images of multilayer FePSe_3_ at different magnifications. C,D) Dark‐field TEM image of ultrathin FePSe_3_@CS NSs and the corresponding elemental mapping images. TEM images of E) FePSe_3_@CS and I) FePSe_3_@CCM NSs. AFM images and corresponding height analysis of F,G) FePSe_3_@CS NSs and J,K) FePSe_3_@CCM NSs. Size distribution of H) FePSe_3_@CS NSs and L) FePSe_3_@CCM NSs.

**Figure 2:**
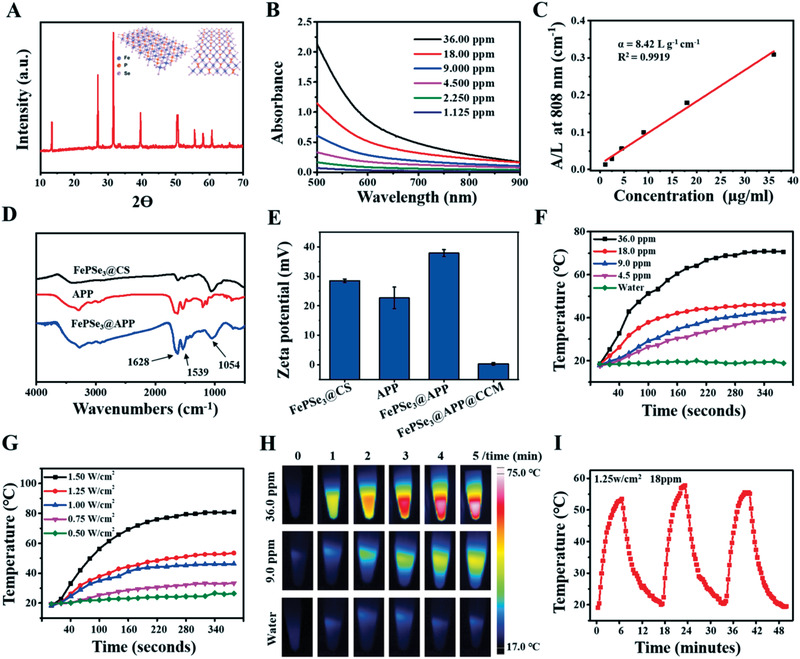
A) XRD spectra and schematic structure of ultrathin FePSe_3_@CS NSs. B) Absorbance spectra of FePSe_3_@CS NSs at varied concentrations. C) Normalized absorbance intensity at *λ* = 808 nm divided by the characteristic length of the cell (*A*/*L*) at varied concentrations. D) FT‐IR spectra of FePSe_3_@CS NSs, APP, and FePSe_3_@APP NSs. E) The zeta potential of APP and these NSs. The temperature curves of FePSe_3_@CS NSs at F) various concentrations and G) varied power densities. H) Typical pictures of thermal imaging. I) Temperature curve of FePSe_3_@CS NSs for three on/off cycles under 808 nm laser irradiation.

The morphology of the bulk FePSe_3_ showed the tightly packed multilayer structure of uniform sheets, which was determined by scanning electron microscopy (SEM; **Figure** **1**A,B). Considering the aggregation of bulk FePSe_3_ in PBS and physiological conditions, CS‐modified FePSe_3_ (denoted as FePSe_3_@CS) was prepared by liquid exfoliation through sonication in CS solution. Figure 1C,D shows the scanning transmission electron microscopy‐dark field (STEM‐DF) images of typical exfoliated FePSe_3_@CS NSs, along with the corresponding energy‐dispersive X‐ray spectroscopy (EDX) elemental mapping, which confirmed the composition of Fe, P, and Se. Furthermore, the FePSe_3_@CS NSs exhibited uniform distribution (Figure 1E) and good stability under physiological conditions for 72 h (Figure S1, Supporting Information). Then, CCM was extracted and extruded as previously reported,^[^ ^45^, ^52^ ^]^ which was used to further coat FePSe_3_@CS NSs. CCM‐camouflaged FePSe_3_@CCM NSs were synthesized by mixing all components together, followed by extrusion through porous polycarbonate membranes. The transmission electron microscopy (TEM) image of FePSe_3_@CCM NSs is shown in Figure 1I. After decoration by CCM, atomic force microscopy (AFM) images proved that the thickness of the NSs had obviously increased from ≈2.5 nm for FePSe_3_@CS to 14.9 nm for FePSe_3_@CCM NSs, which is consistent with the previously reported thickness of a living cancer cell membrane of 12 nm (Figure 1F,G,J,K).^[^ ^28^, ^45^ ^]^ Similarly, the average hydrodynamic size of NSs, measured by dynamic light scattering (DLS), increased from 130 nm for FePSe_3_@CS to 230 nm for FePSe_3_@CCM (Figure 1H,L). These above results verified the successful coating of CCM on the surface of FePSe_3_@CS NSs. The crystalline phase purity of FePSe_3_@CS NSs was confirmed by powder X‐ray diffraction (XRD) (**Figure** **2**A), which is similar to the literature.^[^ ^29^ ^]^ The vis–NIR absorbance spectra of FePSe_3_@CS NSs at various concentrations clearly revealed that the modified NSs exhibited a broad absorption band ranging from vis to NIR regions (Figure 2B), which is similar to traditional 2D layer NSs. The extinction coefficient (*α*), which refers to the light absorption ability of FePSe_3_@CS NSs, was evaluated through the Lambert–Beer law. As shown in Figure 2C, the extinction coefficient of FePSe_3_@CS NSs at 808 nm was calculated to be 8.42 L g^−1^ cm^−1^, indicating the potency of FePSe_3_NSs in PTT application. To obtain the targeted product of FePSe_3_@APP@CCM, we first conjugated the APP to the FePSe_3_@CS surface by a coupling reaction between the —COOH group of APP and the —NH_2_ group of CS through *N*‐hydroxysuccinimide (NHS)/1‐(3‐dimethylaminopropyl)‐3‐ethylcarbodiimide hydrochloride (EDC) conjugation. Fourier transform infrared spectroscopy (FT‐IR) of FePSe_3_@CS and FePSe_3_@APP NSs both exhibited an obvious peak at 1054 cm^−1^ corresponding to the stretching vibration of C—O, which is the characteristic peak of CS (Figure 2D). The characteristic peaks of FePSe_3_@APP at 1628 and 1539 cm^−1^ were attributed to the first and secondary amide groups (CO—NH—), indicating the successful linkage of APP to the surface of NSs. In addition, the change in zeta potential of the NSs was studied to monitor the different surface decorations. Figure 2E shows that the zeta potentials of FePSe_3_@CS, APP, FePSe_3_@APP, and FePSe_3_@APP@CCM were +28.5, +24.0, +37.8, and +0.2 mV, respectively. The decrease in the zeta potential of FePSe_3_@APP@CCM is due to the coating of the negatively charged cancer cell membrane. The loading of APP was estimated to be 3.5 mg APP per 100 mg FePSe_3_@APP@CCM. Furthermore, compared to bulk FePSe_3_, FePSe_3_@APP@CCM NSs exhibited superior stability under physiological conditions (RPMI 1640, RPMI 1640 + 10% FBS and PBS) (Figure S2, Supporting Information). As shown in Figure S3 in the Supporting Information, FePSe_3_@CS NSs and FePSe_3_@APP@CCM NSs both showed a low hemolysis ratio (6.7% and 5.3%, respectively) after co‐incubation with red blood cells (RBCs) for 6 h. The RBCs incubated with these NSs had intact and smooth surfaces without damage to cellular functions, indicating the NSs decorated with CS and wrapped with the CCM can inhibit the interaction between NSs and the RBCs. So, the blood compatibility of FePSe_3_@CS NSs and FePSe_3_@APP@CCM NSs can be significantly improved and an ideal protective effect can be achieved, which indicates that these modified NSs are promising for in vitro and in vivo biomedical applications.

### 2.2 Photothermal Ability of FePSe_3_@CS NSs

To further explore the potential of FePSe_3_@CS NSs as a photothermal agent, we monitored and recorded the photothermal‐heating curves of FePSe_3_@CS NSs and the corresponding thermal images. As shown in Figure 2F,H, the temperature of the FePSe_3_@CS NSs with an elevated concentration increased in a dose‐dependent manner under 808 nm laser irradiation (1.0 W cm^−2^). In contrast, the temperature of pure water showed no obvious change, which suggested that the presence of NSs efficiently converted NIR light into thermal energy. Similarly, the FePSe_3_@CS NSs showed power density‐dependent photothermal conversion efficiency (Figure 2G). The photothermal stability of FePSe_3_@CS NSs was evaluated by testing three recycling temperature variations of the FePSe_3_@CS NS solution where no significant deterioration during recycling was observed (Figure 2I), indicating the potential application of FePSe_3_@CS NSs as PTT agents for cancer treatment. The photothermal conversion efficiency of FePSe_3_@CS NSs was calculated to be 30.4% (Figure S4, Supporting Information), which was comparable to that of traditional 2D NSs.^[^ ^53^, ^54^ ^]^ These results showed that FePSe_3_@CS NSs have a good photothermal conversion ability and lay a firm foundation for further PTT applications.

### 2.3 MRI and PAI Ability of FePSe_3_@CS NSs

**Figure 3:**
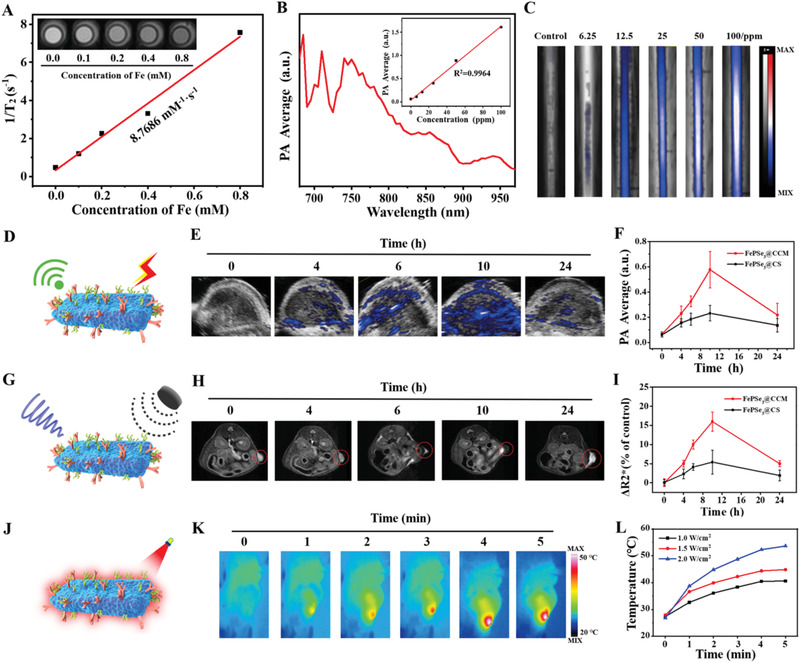
A) T_2_‐weighted MR images and fitted curve of the T_2_‐weighted relaxation rates (*r*_2_) of FePSe_3_@CS NSs with different Fe concentrations ranging from 0.10 × 10^−3^ to 0.80 × 10^−3^ m under 1.5 T magnetic field. B,C) PA spectrum, PA values, and the corresponding PA images of FePSe_3_@CS NSs with different concentrations ranging from 6.25 to 100 ppm. D) Schematic illustration for PAI ability, E) PAI of FePSe_3_@CCM NSs in the tumor site, and F) the corresponding PA value after i.v. injection with FePSe_3_@CCM NSs and FePSe_3_@CS NSs at different time intervals. G) Schematic illustration for MRI ability, H) T_2_‐weighted MR images of FePSe_3_@CCM NSs in the tumor site, and I) the Δ*R*_2_^*^ value in the tumor site after i.v. injection with FePSe_3_@CCM NSs and FePSe_3_@CS NSs at different time intervals. J) Schematic illustration for thermal imaging, K) thermal imaging in the tumor site, and L) the corresponding temperature curves with FePSe_3_@CCM NSs i.v. injection after 10 h at different power densities.

Fe is frequently exploited as a T_2_‐weighted contrast agent because of its capacity to shorten the *T*_2_ relaxation time of its surrounding water protons. As Fe is one of the components of FePSe_3_, FePSe_3_ NSs are potential MRI contrast agents. Therefore, for the first time, we evaluated the T_2_‐weighted performance of FePSe_3_. From T_2_‐weighted MRI, FePSe_3_@CS was found to gradually reduce the MR signal intensity with increasing Fe concentration (**Figure** **3**A). By linearly fitting the *T*_2_ relaxation rate (1/*T*_2_) versus Fe concentration, we found that the relaxivity was 8.77 mm ^−1^ s^−1^, demonstrating that these NSs could be a practical contrast agent for MR imaging. As depicted above, FePSe_3_ NSs exhibited good photothermal properties in the NIR range and were regarded as potential PAI agents; however, the PAI of this type of material has not yet been measured. Therefore, for the first time, we studied the PA phantom of FePSe_3_@CS NSs. As expected, the PA signals of FePSe_3_@CS NSs were significantly enhanced with increasing concentration (Figure 3B,C). A sharp peak at 710 nm appeared in the PA spectrum. Additionally, the PA signal produced by FePSe_3_@CS NSs demonstrated a linear relationship with concentration. These results clearly showed that MRI and PAI can be achieved by the single 2D nanoplatform built from FePSe_3_.

### 2.4 Multimodal Imaging of FePSe_3_@CCM and FePSe_3_@CS NSs In Vivo

To further determine the feasibility of FePSe_3_@CCM and FePSe_3_@CS NSs for multimodal imaging in vivo, we established a xenografted CT26 tumor model in mice, which can also be used to monitor their accumulation at the tumor site. After intravenous administration, PA images and the related signal curves at the tumor site were captured at different time intervals (0, 4, 6, 10, and 24 h). As shown in Figure 3E,F, the PA signal at the tumor site was clearly observed. The average signal of FePSe_3_@CCM NSs gradually increased and reached a peak with a value of 0.576 a.u. at 10 h post injection and then fell to 0.217 a.u. at 24 h. Notably, FePSe_3_@CCM demonstrated an obviously more intensive PA signal than FePSe_3_@CS NSs. A similar trend was observed in the T_2_‐weighted MR images of tumors in Figure 3H,I. The MR signals reached their maximum value (darkest) at 10 h post injection, which then decreased gradually. In addition, FePSe_3_@CCM showed a better contrast than FePSe3@CS, which suggests that camouflaged CCM facilitated accumulation at the tumor site via adherent molecules and surface antigens preserved by CCM on the surface of these NSs. Additionally, the time point at which the maximal accumulation achieved was chosen to perform photothermal imaging. The NIR irradiation was exposed to the tumor site at 10 h post intravenous injection. The thermal images and temperature curves were recorded by a thermal imager. As shown in Figure 3K,L, the temperature of the tumor site showed an obvious upward trend under laser radiation, and the NIR‐heating behaviors of FePSe_3_@CCM NSs were power‐dependent. The temperature could reach 40.6, 44.9, and 53.6 °C under 1.0, 1.5, and 2.0 W cm^−2^ laser irradiation, respectively, further validating its effective accumulation and excellent photothermal conversion at the tumor site, which indicated that FePSe_3_@CCM NSs not only are a feasible contrast agent for thermal imaging but could also be applied for PTT in vivo.

### 2.5 Homologous Target and Immune Escape Ability of FePSe_3_@CCM NSs In Vitro

**Figure 4:**
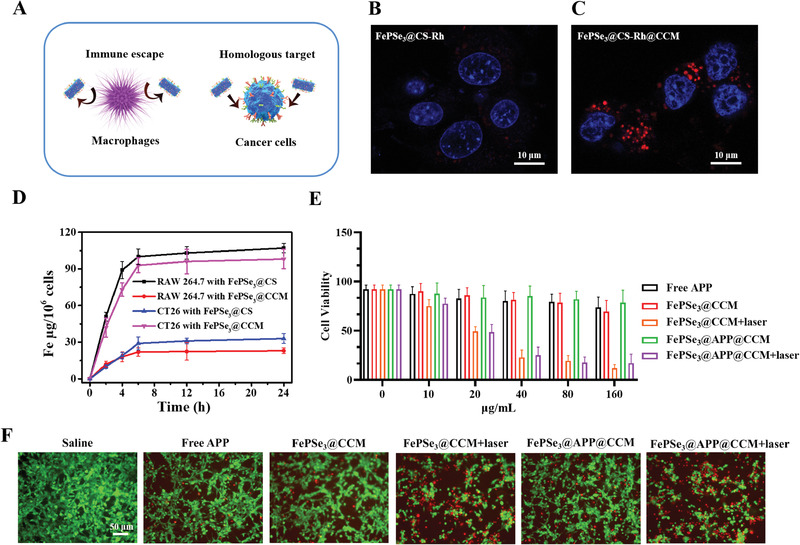
A) Schematic illustration for immune escape and homologous targeting ability of FePSe_3_@CCM NSs. B,C) CLSM images of CT26 cells after incubation with FePSe_3_@CS‐Rh and FePSe_3_@CS‐Rh@CCM NSs for 6 h; scale bar: 10 µm. D) Quantitative analysis of cellular uptake of CT26 and RAW 264.7 cells under different conditions. E) Cell viability of CT26 cancer cells incubated with diverse concentrations of free APP, FePSe_3_@CCM, and FePSe_3_@APP@CCM. For the FePSe_3_@CCM and FePSe_3_@APP@CCM groups, the cells were irradiated with or without an NIR laser (808 nm, 1.5 W cm^−2^, 5 min) after 6 h incubation, followed by further incubation for 18 h. F) Fluorescent images of CT26 cells stained with calcein AM (green) and PI (red) after different treatments; scale bar: 50 µm.

For cancer therapy, it is vital for nanomaterials to be taken up and internalized by cancer cells. To assess the cellular uptake of FePSe_3_@CS and FePSe_3_@CCM, we labeled them with NHS‐rhodamine (NHS‐Rh) (denoted as FePSe_3_@CS‐Rh and FePSe_3_@CS‐Rh@CCM, respectively). CT26 cancer cells were incubated with these two NSs for 6 h and then analyzed by confocal laser scanning microscopy (CLSM, Leica SP8). As shown in **Figure** **4**B,C, the Rh‐modified NSs were mainly located in the cytoplasm. In addition, the fluorescence intensity of FePSe_3_@CS‐Rh@CCM (red) was obviously stronger than that of FePSe_3_@CS‐Rh, indicating the excellent cellular internalization capacity of FePSe_3_@CS‐Rh@CCM NSs.

To further quantitatively determine the cellular internationalization, we incubated CT26 and RAW264.7 cells with FePSe_3_@CS and FePSe_3_@CCM for 2, 4, 6, 12, and 24 h with 100 µg mL^−1^ Fe. Their cellular uptake in RAW 264.7 and CT26 cells was then illustrated and quantified as displayed in Figure 4A,D. The cellular uptake of FePSe_3_@CCM was much lower than that of FePSe_3_@CS in RAW 264.7 cells, which is likely due to the immune escape of CCM‐camouflaged NSs resulting from CCM‐biomimetic surface functionalization. The opposite phenomenon was found in CT26 cells, where FePSe_3_@CCM presented a significantly higher uptake, suggesting that the homologous targeting ability of CCM facilitated the cellular uptake of FePSe_3_@CCM by cancer cells.

### 2.6 Photothermal Therapeutic Effect of FePSe_3_@APP@CCM NSs In Vitro

Encouraged by the efficient cellular uptake in cancer cells and photothermal conversion performance, we measured the cytotoxicity of FePSe_3_@APP@CCM in CT26 cancer cells to verify its photothermal therapeutic effect on tumor ablation in vitro. Upon treatment with FePSe_3_@APP@CCM/FePSe_3_@CCM and exposure to NIR laser irradiation, the cell viability showed a remarkable dose‐dependent decrease (Figure 4E). However, in the absence of laser irradiation, the cell viability showed no obvious decline, demonstrating an efficient photothermal therapeutic effect in vitro.

Furthermore, the calcein AM (AM = acetoxymethyl) and propidium iodide (PI) co‐staining method was used to verify the therapeutic efficacy. The green fluorescence from calcein AM is usually regarded as a signal of living cells, while the red fluorescence from PI represents dead cells. As shown in Figure 4F, cells treated with FePSe_3_@CCM and FePSe_3_@APP@CCM demonstrated no obvious cytotoxicity in the absence of laser irradiation, as shown by the strong green fluorescence. Under laser irradiation, these treated groups exhibited intense red fluorescence, confirming that these NSs are capable of photothermal tumor ablation in vitro.

### 2.7 Immune Response in a Peripheral Blood Mononuclear Cell (PBMC)/CT26 Coculture System

**Figure 5:**
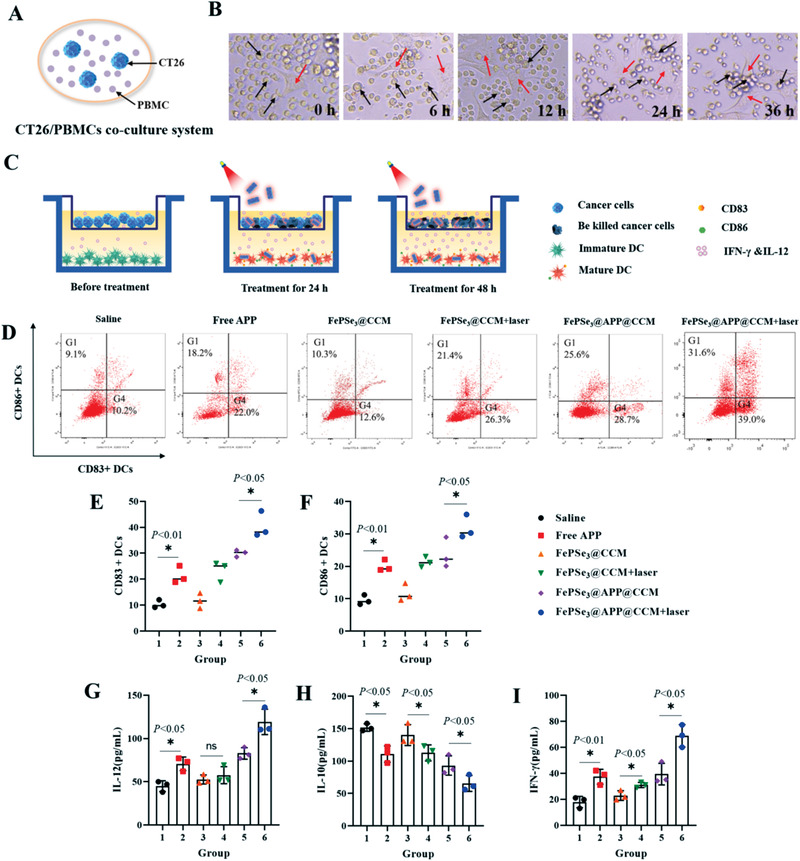
FePSe_3_@APP@CCM NS‐mediated immune response in vitro. A) Schematic diagram for the interaction of CT26/PBMCs in coculture systems. B) Pictures of CT26 cancer cells adhered and killed by PBMCs in coculture systems treated with FePSe_3_@APP@CCM plus laser irradiation after different time points; the red arrows represent CT26 cells, and black arrows represent PBMCs. C) Schematic illustration of the transwell system showing the design of the DC maturation experiment in vitro. CT26 cells were cocultured in the upper chamber, while DCs cells were cultured in the lower chamber. The tumor cells and DCs in the CT26/DC coculture system were treated by different groups for 48 h. D) Flow cytometry plots showing the mature DCs after different treatments in the CT26/DC coculture system. E,F) The amount of CD83+ and CD86+ on the DC surface measured using flow cytometry after 48 h of various treatments in the Transwell CT26/DC coculture system. G–I) Statistical chart of cytokine levels of IL‐12, IL‐10, and IFN‐*γ* in coculture supernatant secreted by DCs after different treatments for 48 h (*n* = 5, mean ± SD; ^*^ *p* < 0.05 and ns = no significance).

PBMCs are monocytes extracted from peripheral blood and are considered precursors of dendritic cells (DCs) and macrophages. PBMCs have the ability to recognize and kill tumor cells by phagocytosis, produce antibodies, participate in the tumor immune response, and could present to T lymphocytes after engulfing the tumor, thereby playing an important role in the body's immune system.^[^ ^55^ ^]^ It has been revealed that PBMCs are closely related to PD‐1 inhibitor immunotherapy. The number of CD14+ and CD16+ PMBCs in blood is the most reliable indicator of the progression‐free survival (PFS) and overall survival of patients after treatment.^[^ ^56^ ^]^ To investigate the effects of these NSs on PBMCs under laser irradiation, we established a PBMC/CT26 coculture system, as shown in **Figure** **5**A. The morphological images of PBMCs and CT26 in the coculture system treated with FePSe_3_@APP@CCM under laser irradiation were captured at 0, 6, 12, 24, and 36 h. As demonstrated in Figure 5B, the red arrows represent CT26 cells, and black arrows represent PBMCs. PBMCs gradually moved toward CT26, followed by recognition, adhesion, and the final engulfment of CT26. The cytotoxicity of CT26 and PBMCs cells in the coculture system was then investigated (Figure S5, Supporting Information). The viability of CT26 cells declined significantly after treatment with FePSe_3_@APP@CCM under laser irradiation. However, PBMCs responded differently toward the treatment. Incubation with free APP and FePSe_3_@APP@CCM resulted in an increase in cell viability, which is likely due to the presence of APP blocking the expression of PD‐1 on monocytes, leading to the reduction of programmed death of PBMCs and enhanced proliferation. Interestingly, NIR laser irradiation was found to have a positive effect on the survival of PBMCs. In conclusion, CT26 cells were killed not only by the PTT therapeutic effect but also by the increased number of PBMCs, suggesting that a synergistic photothermal–immunotherapeutic effect existed in vitro.

### 2.8 FePSe_3_@APP@CCM NSs Enhance DC‐Induced Tumor Immunotherapy in a CT26/DC Coculture System

Previous studies have demonstrated that immature DCs can identify and capture tumor antigens and can be transformed into immunogenic mature DCs by antigen‐derived signals or drugs.^[^ ^57^ ^]^ Mature DCs can secrete cytokines, including IFN‐*γ* and IL‐12, and form co‐stimulatory molecules to activate T cells. These cells could also present tumor antigens to CTLs for cancer immunotherapy in the form of peptides bound to self‐major histocompatibility complex (MHC) complexes.^[^ ^58^ ^]^ Thus, measuring the maturation of DCs can be used to evaluate DC‐related immune responses. To investigate the maturation of DCs induced by FePSe_3_@APP@CCM NSs in vitro, we established a CT26/DC coculture system by using a transwell chamber, as shown in Figure 5C. The DCs were placed in the lower layer, and CT26 cells were placed in the upper layer. The coculture system was treated with saline, free APP, FePSe_3_@CCM, FePSe_3_@CCM + laser irradiation, FePSe_3_@APP@CCM, and FePSe_3_@APP@CCM + laser irradiation. The maturation of DCs was measured by staining and quantifying the expression of CD83+ and CD86+, two typical markers of mature DCs.^[^ ^59^ ^]^ As shown in Figure 5D–F, higher expression of CD83+ and CD86+ was found upon treatment with FePSe_3_@APP@CCM (28.7% and 25.6%), which was higher than that in the saline group (10.2% and 9.1%) and FePSe_3_@CCM group (12.6% and 10.3%), indicating that the encapsulated APP from FePSe_3_@APP@CCM significantly increased the maturation of DCs, thus increasing the DCs‐mediated immune effect. Comparing with the FePSe_3_@CCM group, the expression of CD83+ and CD86+ after treatment with FePSe_3_@CCM plus laser was greatly increased to 26.3% and 21.4%, indicating that the laser irradiation also increased the maturation of DCs. Interestingly, the combination with PTT synergetic treatment increased the expression levels of CD83+ and CD86 in the FePSe_3_@APP@CCM plus laser group to 39.0% and 31.6%, respectively, which were significantly higher than those in the nonirradiated group. The results confirmed that the combined photothermal–immunotherapeutic effect of FePSe_3_@APP@CCM efficiently enhanced the maturation of DCs and DC‐related immune responses.

The secretion of the immune factors IL‐10, IL‐12, and IFN‐*γ* was further investigated to confirm out conclusion. IL‐12 and IFN‐*γ* are known to mediate interactions between DCs and PD‐1 inhibitors. Studies have revealed that anti‐PD‐1 cancer immunotherapy requires T cell‐DC crosstalk where the cytokines IFN‐*γ* and IL‐12 are closely involved.^[^ ^60^ ^]^ In contrast, IL‐10 has been reported to downregulate the expression of MHCII on the surface of DCs and reduce the antigen presentation ability of DCs, thereby inhibiting the activity of T cells.^[^ ^61^ ^]^ In our study, treatment with FePSe_3_@APP@CCM plus laser irradiation significantly upregulated the expression of cytokines IL‐12 and IFN‐*γ*, indicating that their secretion was increased along with DC maturation (Figure 5G,I). However, we found that the secretion of IL‐10 by DCs was obviously inhibited by the treatment, suggesting that the enhanced immune responses of T cells were a result of the reduced IL‐10 expression (Figure 5H). Taken together, upon NIR laser irradiation, FePSe_3_@APP@CCM matured and activated immature DCs, enhanced the secretion of IFN‐*γ* and IL‐12, and decreased the expression and the consequent inhibitory effect of IL‐10 on T cells, resulting in the enhanced immunity of T cells for killing CT26 cancer cells in the coculture system.

### 2.9 In Vivo Antitumor Effects of FePSe_3_@APP@CCM NSs

**Figure 6:**
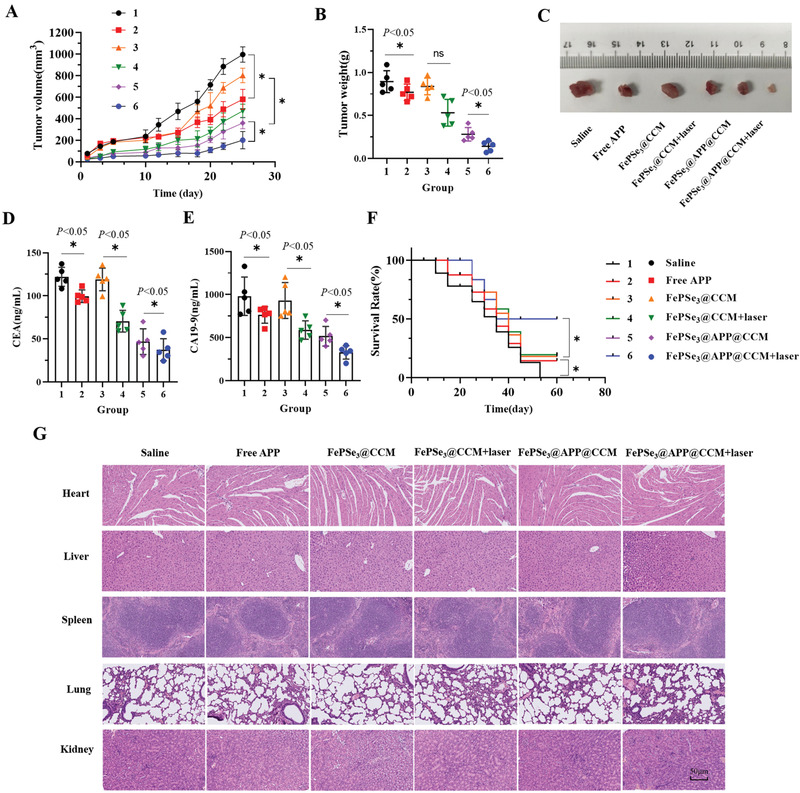
A) Tumor growth curve and B) tumor weight of the tumor‐bearing mice from the different treatment groups (1: control; 2: free APP; 3: FePSe_3_@CCM; 4: FePSe_3_@CCM plus laser irradiation; 5: FePSe_3_@APP@CCM; 6: FePSe_3_@APP@CCM plus laser irradiation). C) Tumor tissues removed from mice after different treatment groups. D,E) Expression of tumor markers CEA and CA19‐9 in the mouse blood serum. F) Survival curve of tumor‐bearing mice treated by different groups. G) Pathological changes detected by H&E staining of the main organs (heart, liver, spleen, lung, and kidney) from mice after receiving intravenous injection with different treatment groups for 25 days. Scale bar: 50 µm (*n* = 5, mean ± SD; ^*^ *p* < 0.05 and ns = no significance).

Encouraged by the in vitro cytotoxicity and immune responses of FePSe_3_@APP@CCM NSs, we assessed their antitumor effects in vivo using the CT26 tumor xenograft model in mice. The tumors showed rapid growth after treatment with saline. The mice treatment with FePSe_3_@CCM (orange line) showed a little effect on suppression the tumor growth comparing to the control group (saline, black line). However, FePSe_3_@APP@CCM (purple line) exhibited significant effect on suppression the tumor growth, indicating the immunotherapy from encapsulated APP. The treatment with free APP (red line) and FePSe_3_@CCM plus laser irradiation (green line) both had growth inhibitory effects, indicating antitumor activity of these treatments alone. However, FePSe_3_@APP@CCM plus laser irradiation notably presented significantly stronger antitumor efficacy compared with that of the other groups (**Figure** **6**A), reflecting the combined effect of PTT and immunotherapy. The tumor weight and images also demonstrated the antitumor effect of various treatment groups (Figure 6B,C). Carbohydrate antigen 19‐9 (CA19‐9) and carcinoembryonic antigen (CEA) are well‐known biomarkers that are highly expressed in numerous cancers, including colorectal cancer, pancreatic cancer, and stomach cancer. Therefore, the expression of these two tumor markers in mouse plasma was further evaluated by ELISAs. As shown in Figure 6D,E, the expression of CEA and CA19‐9 decreased to different extents upon treatment with NSs compared to that in the saline control group, with the most efficient decrease demonstrated by FePSe_3_@APP@CCM plus laser irradiation. Furthermore, the body weight (Figure S6, Supporting Information) and survival rate (Figure 6F) of the mice were also examined. FePSe_3_@APP@CCM plus laser irradiation treatment resulted in the highest survival rate, where 50% of the mice were rescued. The mice treated with saline all died as an effect of tumor aggressiveness; treatment with free APP, FePSe_3_@CCM, FePSe_3_@CCM plus laser irradiation, and FePSe_3_@APP@CCM rescued only ≈20% of mice. The results confirmed that PTT‐immune combined therapy with FePSe_3_@APP@CCM under laser irradiation can efficiently extend the lifespan of mice bearing tumors.

### 2.10 Biosafety Evaluation

The general toxicity of these NSs in vivo was also evaluated. Hematological and histological images of mice intravenously injected with NSs for 25 days were collected and analyzed. The blood biochemical parameters, including alanine aminotransferase (ALT), aspartate aminotransferase (AST), blood urea nitrogen (BUN), creatinine (CRE), lactate dehydrogenase (LDH), and blood platelet (PLT) were obtained and are shown in Figures S7 and S8 in the Supporting Information. No obvious abnormality was observed, indicating that the nanomedicine has no obvious toxicity to the liver, kidney, or other organs. Furthermore, we performed histological analysis of the main organs by Hematoxylin & Eosin (H&E) staining. As shown in Figure 6G, slices of major organs, including the heart, liver, spleen, lung, and kidney, showed no obvious inflammation and exudation or other pathological lesions, demonstrating their biosafety and low toxicity to normal tissues as nanomedicine for cancer imaging and therapy.

### 2.11 FePSe_3_@APP@CCM NSs Downregulate PD‐1 Expression

**Figure 7:**
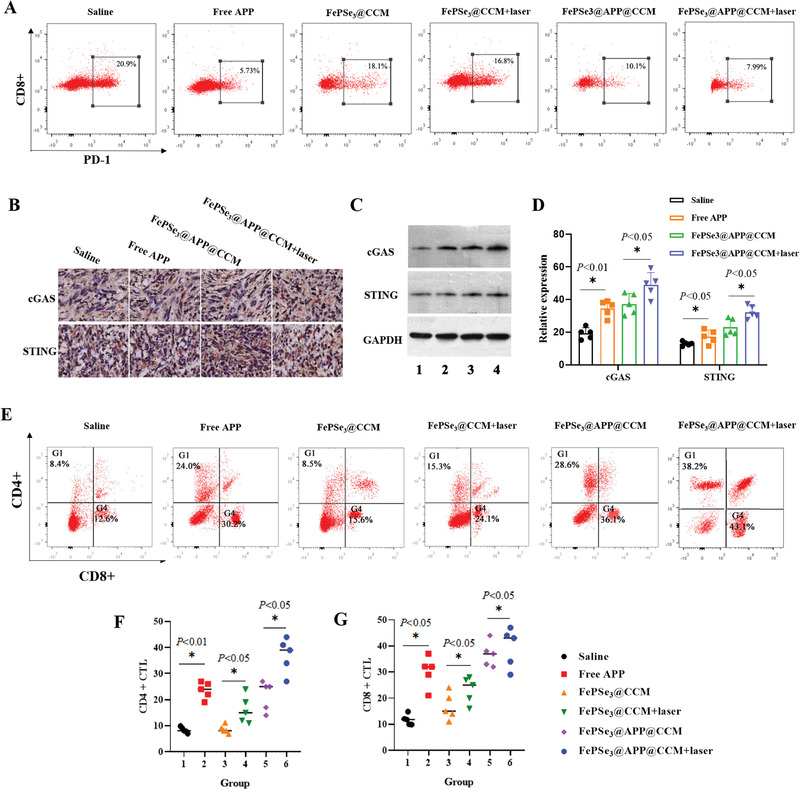
A) Flow cytometric quantification of PD‐1 expression on splenic lymphocytes isolated from CT26 tumor‐bearing C57BL/6J mice treated with different groups (1: control; 2: free APP; 3: FePSe_3_@CCM; 4: FePSe_3_@CCM + laser irradiation; 5: FePSe_3_@APP@CCM; 6: FePSe_3_@APP@CCM + laser irradiation). Expression of the cGAS–STING signaling pathway in tumor sites assayed by B) immunohistochemistry and C) western blots. D) Statistical chart of the protein expression of cGAS and STING. E) Flow cytometry showing different groups of activated CD4+ and CD8+ CTLs in splenic lymphocytes. F,G) Statistical chart of CD4+ and CD8+ CTLs based on flow cytometry image data in (E) from different treatment groups in vivo (*n* = 5, mean ± SD; ^*^ *p* < 0.05 and ns = no significance).

PD‐1 is overexpressed during cancer or inflammation;^[^ ^12^, ^16^ ^]^ thus, the expression of PD‐1 can be used to predict immunosuppression. High expression of PD‐L1 in CT26 tumor cells was measured and confirmed using immunofluorescence assay (Figure S9A, Supporting Information) and western blotting (Figure S9B, Supporting Information). To evaluate the effect of APP blocking PD‐1 protein on PBMCs in vivo, we detected the expression of PD‐1 on PBMCs isolated from CT26 tumor‐bearing mice by flow cytometry. As shown in **Figure** **7**A and Figure S10 in the Supporting Information, PD‐1 expression was significantly downregulated to 5.73%, 10.1%, and 7.99% after treatment with free APP, FePSe_3_@APP@CCM, and FePSe_3_@APP@CCM + laser irradiation, respectively. Treatment with saline, FePSe_3_@CCM, and FePSe_3_@CCM plus laser irradiation did not cause an obvious change in PD‐1 expression. Overall, the presence of APP downregulated PD‐1 expression in PBMCs. Imaging showed that the alleviated expression of PD‐1 can decrease its interaction with its ligand molecules PD‐L1 and PD‐L2, causing a negative effect on the immunosuppressive responses mediated by the PD‐1 pathway and consequently enhanced immune responses.

### 2.12 Immune Response In Vivo

Cancer immunotherapy utilizes the innate immune system to recognize, attack, and ablate tumor cells and ultimately prolong overall survival. Antitumor effects in vivo suggested that in combination with PTT directly killing tumor cells, FePSe_3_@APP@CCM plus laser irradiation loaded with anti‐PD‐1 peptide and PTT‐induced immunotherapy can efficiently inhibit tumor growth. To investigate whether therapeutic effects in vivo are related to the activation of immunological reactions, we studied the NS‐induced immune responses in CT26 tumor‐bearing mice. First, flow cytometry was employed to assay the expression of CD83+ and CD86+ on DCs extracted from mouse spleens to reflect the ability of FePSe_3_@APP@CCM to promote the maturation of DCs in vivo. As expected, the expression of CD83+ and CD86+ DCs was enhanced, with FePSe_3_@APP@CCM plus laser irradiation treatment showing the greatest enhancement (Figure S11A–C, Supporting Information). We further observed the increased expression of DC‐secreted cytokines, including IFN‐*γ* and IL‐12, while the level of IL‐10 was found to be decreased. In addition, the levels of IFN‐*γ* and IL‐12 in serum upon treatment with FePSe_3_@APP@CCM plus laser irradiation were much higher than those in the other groups. These observations further validated the immune responses in vivo by FePSe_3_@APP@CCM under laser irradiation (Figure S11D–F, Supporting Information).

Previous studies also showed that activation of the cyclic GMP‐AMP synthase (cGAS)–stimulator of interferon genes (STING) signaling pathway plays an important role in both the activation of DCs and the apoptosis of tumors. The cGAS enzyme senses the presence of abnormal DNA in the cytoplasm (these abnormal DNA molecules are related to tumor occurrence), activates the STING protein, promotes DC maturation, and finally triggers tumor immunity.^[^ ^62^ ^]^ Immunohistochemistry and western blotting were then applied to detect the cGAS–STING signaling pathway after treatment with NSs. As shown in Figure 7B–D, the obvious upregulation of the cGAS–STING signaling pathway was observed, suggesting that NSs enhanced IFN‐*γ* production and DC maturation probably through activating the cGAS–STING signaling pathway. Additionally, treatment with FePSe_3_@APP@CCM plus laser irradiation showed the most efficient upregulation of the cGAS–STING signaling pathway, indicating that the PTT effect has a role in promoting the cGAS–STING signaling pathway and subsequent tumor immunity.

The effect of FePSe_3_@APP@CCM NSs on the activation of tumor immune effector T cells (CTLs) was further tested using flow cytometry. CTLs are the ultimate effector T cells of tumor immunity and can directly kill all types of cancer cells. In tumorigenesis, immunologic tolerance within the tumor microenvironment leads to the dysfunction and exhaustion of CTLs.^[^ ^63^ ^]^ Therefore, quantifying CTLs can reflect the therapeutic effects. As shown in Figure 7E–G, after treatment with FePSe_3_@APP@CCM under laser irradiation, the expression of CD4+ was found to be 38.2%, which was higher than that after incubation with saline (8.4%), FePSe_3_@CCM (8.5%), FePSe_3_@CCM plus laser irradiation (15.3%), and FePSe_3_@APP@CCM (28.6%). Similarly, the expression of CD8+ T cells by FePSe_3_@APP@CCM under laser irradiation was 43.1%, which was significantly higher than that of the other groups. We also noted that the expression of CD4+ and CD8+ by FePSe_3_@APP@CCM was similar to that after treatment with free APP, which was higher than the expression after FePSe_3_@CCM treatment, suggesting that CD4+ and CD8+ expression was enhanced by APP. In addition, after treatment with FePSe_3_@CCM plus laser irradiation, the expression of CD4+ and CD8+ was higher than that after FePSe_3_@CCM alone, indicating that PTT had a positive effect on CD4+ and CD8+ expression and their related immune responses. Based on the analysis, we demonstrated that CD4+ and CD8+ T cells were reactivated by immunotherapy with an NIR laser and APP, thereby alleviating the exhaustion of CTLs. The results indicated an improved immune response via the combination of multiple immunotherapies and PTT mediated by FePSe_3_@APP@CCM plus laser irradiation, which led to significantly increased antitumor efficiency.

## 3 Conclusion

In conclusion, we developed a multifunctional 2D ultrathin FePSe_3_@APP@CCM nanosystem with a multimodel imaging ability, photothermal performance, and PD‐1 blocking capability. The functionalized 2D FePSe_3_‐based NSs have an efficient tumor targeting ability because of the CCM decoration and could facilitate precise monitoring in the tumor region via MRI and PAI. The in vivo MRI and PAI revealed the preferential localized accumulation of FePSe_3_@CCM in the tumor site and reaching a maximum accumulation at 10 h post injection, which potentially can be used for in situ cancer diagnosis in deep tissues. In addition, NSs can be applied for NIR‐induced PTT to cause direct damage to cancer cells, trigger DC maturation and related cytokine secretion to activate T cell‐related immune responses, and achieve immunotherapy with a PD‐1 checkpoint blockade strategy by blocking the PD‐1/PD‐L1 pathway. Therefore, the FePSe_3_@APP@CCM NSs could achieve efficient synergistic anticancer PTT and immunotherapy in vitro and in vivo, showing low toxicity but a strong immune response and thus successfully prolonging the lifespan of xenografted mice. We strongly believe our strategy provides a valuable example for broadening MXP_3_‐based NSs in biomedical applications.

## 4 Experimental Section

##### Materials

FePSe_3_ powder was purchased from SixCarbon Technology Shenzhen. The APP (sequence: (SNTSESF)_2_KFRVTQLAPKQIKE‐COOH) was purchased from GL Biochem (Shanghai) Company. CS, NHS, EDC, PI, calcein‐AM, Hoechst 33342, and NHS‐rhodamine were purchased from Sigma‐Aldrich Company. All cell culture media were purchased from Thermo Fisher. Milli‐Q water was applied in all experiments.

##### Cell Lines and Animals

CT26 cells and RAW 264.7 cells were purchased from FuHeng Biology Co., Ltd. (Shanghai, China). PBMCs used for coculture were extracted from the blood of tumor‐bearing mice. The DCs used for coculture were induced from PBMCs using rhGM‐CSF, rhIL‐4, and rhTNF‐*α* (PeproTech Corporation, USA) for 7 days. These CT26 cells, RAW 264.7 cells, PBMCs, and DCs were all cultured as previously reported.^[^ ^6^, ^16^, ^45^ ^]^ These 18–22 g C57BL/6J mice were obtained from the Institute of Zoology, Chinese Academy of Sciences (Beijing, China). All animal procedures were carried out under the guidelines approved by the Hong Kong Polytechnic University Animal Study Committee.

##### Synthesis of FePSe_3_@CS NSs

FePSe_3_ powder (50 mg) was weighed and ground into finer powder in a mortar. Then, the ground powder was dissolved in 50 mL of CS (0.8 mg mL^−1^) and exfoliated via sonication for 24 h. After sonication, the excessive CS was removed by centrifugation at 12 000 rpm for 30 min. Finally, the ultrathin CS‐modified FePSe_3_ NSs (FePSe_3_@CS) were obtained from the supernatant by centrifugation at 1500 rpm for 60 min.

##### Synthesis of FePSe_3_@APP@CCM NSs

Briefly, APP (25 mg) was dissolved in Milli‐Q water and stirred with NHS (5 mg) and EDC (5 mg) for 2 h. Then, the FePSe_3_@CS NSs (10 mL) were added to the above mixed solution and further stirred for 8 h. Finally, the excess APP was eliminated by dialysis for 24 h to obtain FePSe_3_@APP NSs. The preparation of CT26 CCM vesicles was conducted in a previous report.^[^ ^45^, ^46^, ^52^ ^]^ To coat CCM onto the nanosheets, 1 mL of PBS containing 50 µg FePSe_3_@APP was mixed with the prepared CCM vesicles. After being subsequently extruded 11 times through 200 nm pores, FePSe_3_@APP@CCM NSs were obtained after centrifugation and rinsing, and finally suspended in PBS (pH 7.4) solution for further experiments.

##### Synthesis of FePSe_3_@CS‐Rh and FePSe_3_@CS‐Rh@CCM NSs

The conjugation of FePSe_3_@CS with NHS‐Rh was performed by stirring FePSe_3_@CS NSs (1 mg mL^−1^, 10 mL) and NHS‐Rh (0.5 mg) for 6 h at room temperature in the dark and then dialyzing for 3 days to remove the excess NHS‐Rh to obtain FePSe_3_@CS‐Rh NSs. Then, the FePSe_3_@CS‐Rh NSs were coated by CCM to produce FePSe_3_@CS‐Rh@CCM NSs using the abovementioned protocol.

##### Characterization of FePSe_3_@APP@CCM NSs

The morphology, size, distribution, zeta potential, and thickness of the NSs were characterized by STEM (Jeol JEM‐2100F), SEM (TESCAN VEGA3), AFM (SPM8 Bruker NanoScope 8), and Zetasizer particle size analysis (Malvern Instruments, Limited). In addition, an FT‐IR spectrometer (Thermo Scientific Nicolet IS50), XRD (Rigaku SmartLab 9 kW), and UV–vis spectroscopy (Agilent Cary 8454) were applied to verify the chemical characteristics and structure. The amount of Fe in NSs was quantified by inductively coupled plasma optical emission spectrometry (ICP‐OES, Agilent 710 Series). A NanoOrange Protein Quantitation Kit was used to quantify the amount of APP loaded on the NSs.

##### Hemocompatibility of FePSe_3_@APP@CCM NSs

FePSe_3_@CS (0.5 mL) and FePSe_3_@APP@CCM (0.5 mL) were incubated with 0.5 mL of RBCs in a 37 °C water bath for 0–6 h. Negative control: 0.5 mL of PBS was co‐incubated with 0.5 mL of RBCs. Positive control: 0.5 mL of Triton X‐100 (10 g L^−1^) was incubated with 0.5 mL of RBCs. At the end of culture, the mixed solution was centrifuged at 3000 rpm for 10 min, and the supernatant was collected and placed in a 96‐well plate. The absorbance value of these solutions was detected at 540 nm with spectrophotometry. The morphology of RBCs after 6 h of treatment in different treatment groups was observed by optical microscopy

(1) $$\begin{array}{c} \mathrm{Hemolysis}\left(\%\right)=\frac{A_{\mathrm{sample}}\;-\;A_{\mathrm{NC}}}{A_{\mathrm{PC}}\;-\;A_{\mathrm{NC}}}\times 100\% \end{array}$$

where *A*_sample_ is the absorbance of the sample at 540 nm, *A*_NC_ is the absorbance of the negative control at 540 nm, and *A*_PC_ is the absorbance of the positive control at 540 nm.

##### Photothermal Effects of FePSe_3_@CS NSs

For measurement of the photothermal performance, FePSe_3_@CS NSs with different concentrations (0, 4.5, 9.0, 18.0, and 36.0 ppm) were irradiated by an NIR laser (808 nm, 1 W cm^−2^, 6 min). In addition, FePSe_3_@CS NSs (18.0 ppm) were exposed to an NIR laser with elevated power densities (0.5, 0.75, 1.0, 1.25, and 1.5 W cm^−2^, 6 min). Furthermore, the photothermal stability of FePSe_3_@CS NSs at a concentration of 18.0 ppm was also measured. The aqueous solution was irradiated by an NIR laser (1.25 W cm^−2^) until reaching its maximum temperature, and then, the temperature of the suspension naturally decreased to room temperature. The cycle of heating and cooling was repeated three times, and a thermal imager (Fluke Ti450 IR fusion technology) was used to record these temperature changes and thermal images. The PTT conversion efficiency (*ŋ*) of FePSe_3_@CS NSs was calculated based on the above temperature changes, and the details of the experimental details and calculations are shown in Figure S3 in the Supporting Information.

##### PA and MR Imaging of FePSe_3_@CS NSs in Solution

The PA spectrum of the FePSe_3_@CS NSs and its PA imaging of individual tube phantoms at a wavelength of 710 nm injected with different concentrations of NSs (0, 6.25, 12.5, 25, 50, and 100 ppm) were both monitored by a Fujifilm Visual Sonics Vevo LAZR PA imaging system. The system includes a flash lamp pumped Q‐switched Nd:YAG laser with OPO that is capable of operating from 680 to 970 nm with a peak pulse energy of 26 mJ (at 20 Hz), and transducer LZ250 (13–24 MHz) was selected to conduct the PAI experiment. For in vitro T_2_‐weighted MRI evaluation, the FePSe_3_@CS NSs at elevated Fe concentrations (0 × 10^−3^, 0.1 × 10^−3^, 0.2 × 10^−3^, 0.4 × 10^−3^, and 0.8 × 10^−3^ m) were measured by a 1.5‐T Signa HDxt superconductor clinical MR system (GE Medical System) equipped with a human eight‐channel wrist coil.

##### Multimodal Imaging In Vivo

For in vivo T_2_‐weighted MR dynamic imaging and PA imaging evaluation, mice with CT26 xenograft tumors were established. T_2_‐weighted MR and PA imaging of tumor sites were captured before and after the intravenous injection of FePSe_3_@CS or FePSe_3_@CCM (1 mg mL^−1^, 200 µL). The PA and MR signals and corresponding images of these NSs accumulated in the tumor areas were captured for 24 h. T_2_‐weighted MR dynamic imaging was captured by a Bruker 9.4T high field small animal MR imaging system (Bruker Corporation, USA). The ability of photothermal imaging in vivo was also determined by thermal imaging at 10 h after tail intravenous injections.

##### Immune Response and Cytotoxicity in PBMCs and CT26 Cells in a Coculture System

The PBMC/CT26 coculture system was built by mixing these two types of cells at a ratio of 10:1. PBMCs and CT26 cells were labeled with PKH67. These mixed cells were then treated with free APP, FePSe_3_@CCM, FePSe_3_@CCM + laser irradiation, FePSe_3_@APP@CCM, and FePSe_3_@APP@CCM + laser irradiation for 36 h. For PTT treatment, the cells were irradiated with an NIR laser (808 nm, 1.5 W cm^−2^, 5 min) after 6 h incubation, followed by a further incubation for 30 h. After treatment, the viability of PBMCs and CT26 in this coculture system was determined (details are shown in the Supporting Information). For images, the morphology of PBMCs/CT26 cells at 0, 6, 12, 24, and 36 h was observed by optical microscopy.

##### DCs Maturation and Differentiation In Vitro

A Corning Transwell plate coculture system was constructed to explore the activation and maturation of DCs. While DCs were induced for 7 days, CT26 cells and DCs were cultured in the upper layer and in the lower layer, respectively. This coculture system received various treatments, including APP alone, FePSe_3_@CCM, FePSe_3_@CCM + laser irradiation, FePSe_3_@APP@CCM, and FePSe_3_@APP@CCM + laser irradiation (dose: 0.525 µg mL^−1^ APP and 15 µg mL^−1^ NSs). For PTT treatment, the cells were exposed to irradiation (808 nm, 1.5 W cm^−2^, 5 min) after 6 h incubation, followed by a further incubation for 42 h. After 48 h incubation, DCs were collected and stained with antibodies against CD83+ (CD83 monoclonal antibody, PE‐Cy5, eBioscience, Invitrogen Corporation, USA) and CD86+ (CD86 monoclonal antibody, APC, eBioscience, Invitrogen Corporation, USA) for flow cytometry analysis. An ELISA kit (Meilian Bio Corporation, Shanghai) was used to assay the cytokines such as IL‐12, IL‐10, and IFN‐*γ* released by DCs in cell culture supernatants.

##### Anticancer Effects and Biosafety In Vivo

The in vivo CT26 tumor‐bearing model was constructed by subcutaneously injecting 1 × 10^6^ CT26 breast cancer cells into the right hind limb of mice. Then, these mice were randomly assigned to six groups (*n* = 5) and intravenously injected with saline, free APP, FePSe_3_@CCM, FePSe_3_@CCM + laser irradiation, FePSe_3_@APP@CCM, and FePSe_3_@APP@CCM + laser irradiation (dose: 0.35 mg kg^−1^ APP and 10 mg kg^−1^ NSs) via their tail vein every other day ten times. Particularly, the tumor tissues from the FePSe_3_@CCM + laser and FePSe_3_@APP@CCM + laser groups were irradiated by NIR laser at 10 h after tail vein injection (1.5 W cm^−2^, 10 min). The body weight and tumor volume were measured and recorded for 25 days. After 25 days of treatment, the mice were sacrificed, their blood, tumor tissues, and main organs were collected, and the tumor weights were recorded. In addition, ELISA was used to detect the tumor markers CEA and CA19‐9 and cytokines such as IL‐12, IL‐10, and IFN‐*γ* in blood serum. Immunohistochemistry was used to determine the protein expression of the cGAS–STING signaling pathway. The rest of the tumor tissues were used to extract total protein, and then, the protein expression of the cGAS–STING pathway was measured by Western blots. Mononuclear cells were extracted from mouse spleens and induced into DCs using rhGM‐CSF, rhIL‐4, and rhTNF‐*α* for 7 days, and then, flow cytometry was applied to detect biomarkers of DC maturity, such as CD83+ and CD86+. Finally, the CD4+ and CD8+ CTLs were determined by flow cytometry (CD4 Monoclonal Antibody FITC, CD8a Monoclonal Antibody, APC, eBioscience, Invitrogen Corporation, USA). In addition, the serum biochemical indices were analyzed from mice treated with different groups by i.v. injection, and other main organs were fixed in 4% paraformaldehyde, and then, H&E staining was used to observe the pathological changes.

##### Statistical Analysis

All of the statistical calculations were performed using Origin 9, GraphPad Prism 8, and SPSS 22.0 software. The measurement data were expressed as the mean ± SD, and differences among groups were compared with one‐way analysis of variance (ANOVA), followed by Fisher's LSD for multiple comparisons. *p* < 0.05 was considered statistically significant and ns presented no significance. Kaplan–Meier survival curves were used to analyze mouse survival.

## Conflict of Interest

The authors declare no conflict of interest.

## Supporting information

Supporting Information
