# Supplementary material for: Biomimetic Anti‐PD‐1 Peptide‐Loaded 2D FePSe3 Nanosheets for Efficient Photothermal and Enhanced Immune Therapy with Multimodal MR/PA/Thermal Imaging
Source: Adv Sci (Weinh). 2020 Nov 25;8(2):2003041. doi: 10.1002/advs.202003041 (PMC7816711; doi:10.1002/advs.202003041)
Supplement: Supplementary file 1 — Supporting Information [file ADVS-8-2003041-s001.pdf]

© 2020 Wiley-VCH GmbH

ADVANCED SCIENCE
Open Access

Supporting Information

for *Adv. Sci.*, DOI: 10.1002/advs.202003041

Biomimetic anti-PD-1 peptide-loaded 2D $FePSe_3$ nanosheets for efficient photothermal and enhanced immune therapy with multimodal MR/PA/thermal imaging

Xueyang Fang, Xianlin Wu, Zhendong Li, Lijun Jiang, Wai-Sum Lo, Guanmao Chen, Yanjuan Gu*, and Wing-Tak Wong**

Supporting Information

Title：Biomimetic anti-PD-1 peptide-loaded 2D $FePSe_3$ nanosheets for efficient photothermal and enhanced immune therapy with multimodal MR/PA/thermal imaging

Xueyang Fang, Xianlin Wu, Zhendong Li, Lijun Jiang, Wai-Sum Lo, Guanmao Chen, Yanjuan Gu*, Wing-Tak Wong**

Methods:

Calculation of Photothermal Conversion Efficiency: Briefly, a series concentrations of $FePSe_3$@CS nanosheet were irradiated under different power of 808 nm laser and the temperature fluctuation was monitored by thermal imager.

According to previous reports [1,2], the photothermal conversion efficiency (η) can be calculated based on the following equation (1)

$$\eta = \frac{hS(T_{max} - T_{surr}) - Q_{Dis}}{I(1 - 10^{-A_{808}})} \quad (1)$$

where h is the heat transfer coefficient, S is the surface area of the container, T_{max} is the maximum equilibrium temperature, T_{surr} is ambient temperature of the surroundings, Q_{Dis}

represents the heat dissipated from light absorbed by the quartz sample cell itself, I is the laser power, A_{808} is the absorbance of the nanosheets at the wavelength of 808 nm.

In Equation (1), hS is unknown for calculation. Therefore, we herein introduce sample system time constant τs and a dimensionless driving force temperature θ.

$$\tau_s = \frac{\sum_i m_i C_{p,i}}{hS} \quad (2)$$

$$\theta = \frac{T - T_{surr}}{T_{max} - T_{surr}} \quad (3)$$

where m_i is mass and $C_{p,i}$ is specific heat capacity

According to energy conservation law, Equation (3) could be changed to

$$\frac{d\theta}{dt} = \frac{1}{\tau_s}\left[\frac{Q_{laser}}{hS(T_{max} - T_{surr})} - \theta\right] \quad (4)$$

When the laser is off, Q_{laser} = 0, Equation (4) could be changed into

$$dt = -\tau_s \frac{d\theta}{\theta} \quad (5)$$

$$t = -\tau_s \ln\theta \quad (6)$$

Then, hS can be determined by applying the linear time data from the cooling period versus – ln θ. Substituting hS value into Equation (1), the photothermal conversion efficiency (η) of $FePSe_3$@CS nanosheet at 808 nm could be calculated.

The constant of heat transfer from the system was determined to be τs =175 s of 808 nm. In addition, the m was 0.2 g and the $C_{p,i}$ was 4.2 J/g. Then, hS can be determined by applying the linear time data from the cooling period versus – ln θ as shown in Figure S3. Substituting hS value into Equation (1), the photothermal conversion efficiency (η) of $FePSe_3$@CS nanosheet at 808 nm could be calculated to be 30.4 %.

*In vitro cellular experiments:*To investigate the intracellular uptake of NSs, CT26 cells were seeded into a glass-bottom cell culture dish, which were cultured at 37 ^oC under 5 % CO_2 atmosphere. Then, 50 μLof $FePSe_3$@CS-Rh and $FePSe_3$@CS-Rh@CCM solution was added into the dishes and incubation for 6 h. Afterward, the culture medium was removed and cells were stained with Hochest33342, washed with PBS for three times. Fresh medium was added into each dish and cells were captured by confocal laser scanning microscope (CLSM, Leica SP8) under Ex/Em wavelength at 552/575 nm. To further quantitatively determine the cellular internationalization, CT26 and RAW264.7 cells were incubated with $FePSe_3$@CS and $FePSe_3$@CCM NSs for 2, 4, 6, 12, 24 h at the equivalent Fe concentration of 100 μg/mL. After incubation, the cells were washed gently with PBS for three times, collected and digested with aqua regia and then dilute with 1% HNO_3. The amount of Fe inside cells was measured by inductively coupled plasma-mass spectrometry (ICP-MS).

In vitro photothermal ablation of cancer cells: As for cytotoxicity experiment in vitro, CT26 cells were seeded into 96-well plate at a density of $5x10^4$ cells/well. After incubation with diverse concentration of various formulations (free APP, $FePSe_3$@CCM, $FePSe_3$@CCM plus laser, $FePSe_3$@APP@CCM and $FePSe_3$@APP@CCM plus laser) for 24 h, the cell viability were determined by CCK-8 assay following the standard protocol (Cell Counting Kit, Beyotime Institute of Biotechnology, Shanghai, China). For the NIR mediated cytotoxicity, the cells were irradiated with NIR laser (808 nm, 1.5 W/cm^2, 5 min) after 6 h incubation, followed by a further incubation for 18 h. The data are expressed as the percentage of surviving cells, and reported values are the mean values of three measurements. The CT26 cells were also seeded in 6-well plate and treated them using the same irradiation condition and the dose at 0.525 μg/ml APP and 15 μg/ml NSs. After removed the culture medium, the cells were washed three times with PBS (pH7.4) and then stained with calcein AM (green)

and propidium iodide (PI) (red) to evaluate the cell viability. The fluorescence images of stained cells with different treatments were observed by fluorescence microscopy (ZEISS Observer, Z1).

CT26/PBMCs viability assayed by PKH67: PKH67 kit was used to assay the viability of PBMC and CT26 in co-culture system, and evaluate the killing effect of PBMC on tumor cells. Collect CT26 cells at logarithmic growth stages and prepare cell suspension to 1×10^7 per well. Dilute the PKH67 kit 10 times to prepare working- liquors and add 100μL PKH67 working-liquors to each well. Incubate the labelled cells at room temperature for 30 min and then co-cultured with PBMCs at a ratio of 1:10 in 6-well plate. After treated with different drugs and laser irradiation for 36 h, collect cells and fixed by 5% paraformaldehyde, resuspend cells with PBS, centrifuged cells and then used flow cytometry to detect the viability of CT26. Repeat the above protocols to detect the viability of $PBMC_S$.

DCs induced from PBMCs in vitro: Heparin anticoagulant peripheral blood was diluted with equal volume of cultured medium. Then, add the diluted peripheral blood to lymphocyte separation medium slowly, and centrifuge with 2000 rpm at room temperature for 20 min. After centrifugation, the grey-white anvil membrane cells at the junction of upper and middle layer were PMBCS. After suction with a pipette, $PMBC_S$ was centrifugally purified by adding PBS. The purified PBMCs culture in complete cultured medium at 37 °C for 6 h and then discard nonadherent cells. Then, add culture medium containing cytokines of 1000 U/mL rhGM-CSF and 1000U/mL rhIL-4 in the above purified PBMCs and cultured in 37℃, 5% CO_2 incubator. Replaced half the culture medium every three days and supplement cytokines to original concentration for six days, and then add 200 U/mL TNF-α to stimulate for 24 h. After a total of 7 days of induction, PMBCs were induced into DCs successfully.

PD-L1 expression on CT26 cells assayed by immunofluorescence: PD-L1 expression on CT26 cells was measured using immunofluorescence. Use 4% paraformaldehyde to fix CT26 cells grow on coverslips for 15min, and then these cells were blocked by PBS containing 0.5% fetal bovine serum for 30min in room temperature. Suck off the blocking solution and then add the diluted primary antibody against PD-L1 at 4°C overnight. Next, the coverslips were washed by PBST thrice and then added the fluorescent second antibody, incubated for 1 hour at 37 ℃ in darkroom. Finally, washed them byPBST thrice again, suck off the PBST and then sealed by fluorescent quenching agent, and observed by fluorescence microscope.

PD-1 expression on PMBCs of CT26 tumor-bearing mice assayed by flow cytometry: Mice spleen obtained from different groups after mice executed. Spleen was extruded on a 100 wells stainless steel net and then pre-cooled to prepare cell suspension. The suspension diluted with equal volume of cultured medium and added to lymphocyte separation medium slowly, and then centrifuged with 2000 rpm at room temperature for 20 min. After centrifugation, the grey-white anvil membrane cells at the junction of upper and middle layer were PMBCs. PMBCs were labeled by PD-1 antibody (CD279 Monoclonal Antibody, PE, eBioscience^{TM}, Invitrogen Corporation, USA), and assayed by flow cytometry.

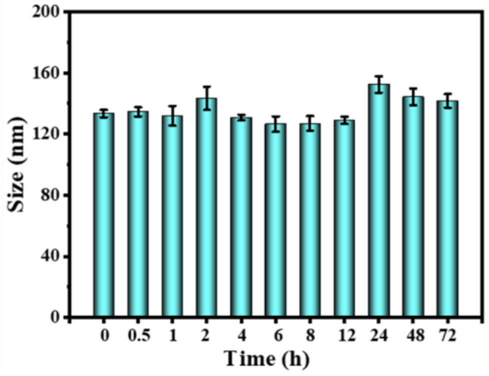

Figure S1. The particle size of $FePSe_3$@CS NSs in H_2O for 72 h.

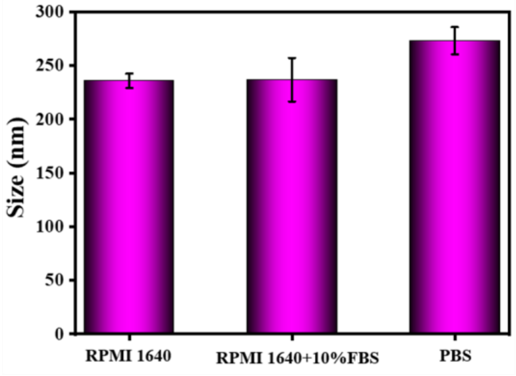

Figure S2. The particle size of $FePSe_3$@APP@CCM NSs in different media.

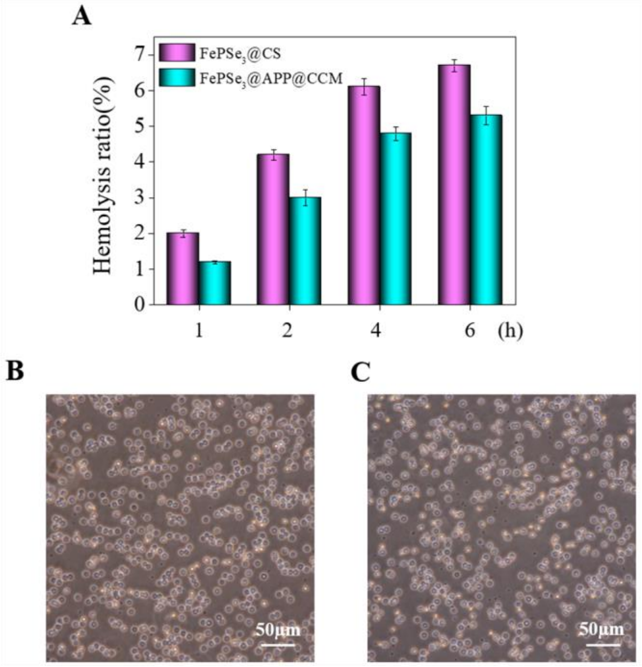

Figure S3. (A) The hemolysis rate of $FePSe_3$@CS NSs and $FePSe_3$@CCM NSs for 24 h; the microscopy image of red blood cells treated with $FePSe_3$@CS NSs (B) or $FePSe_3$@CCM NSs (C) for 6 h.

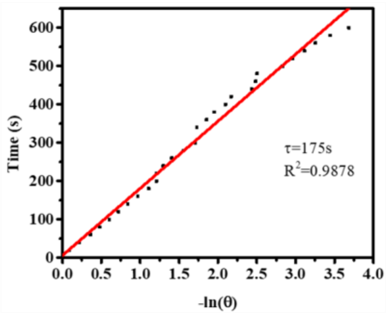

Figure S4. Plot of cooling period versus negative natural logarithm of driving force temperature for evaluating photothermal conversion efficiency.

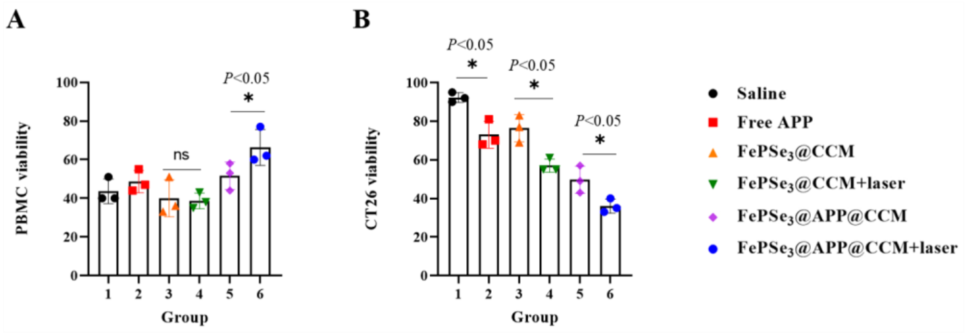

Figure S5. PBMC and CT26 cell viability determined by PKH67 staining and flow cytometry in the CT26/PBMC coculture system after different treatments.(n=3, mean±SD; **P*<0.05 and ns=no significance)

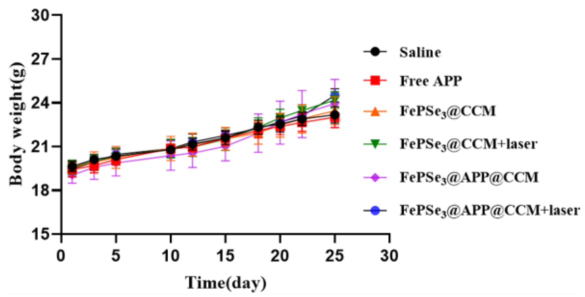

Figure S6. The tumor weight of mice from different treatment groups for 25 days. (n=5, mean±SD; ns=no significance)

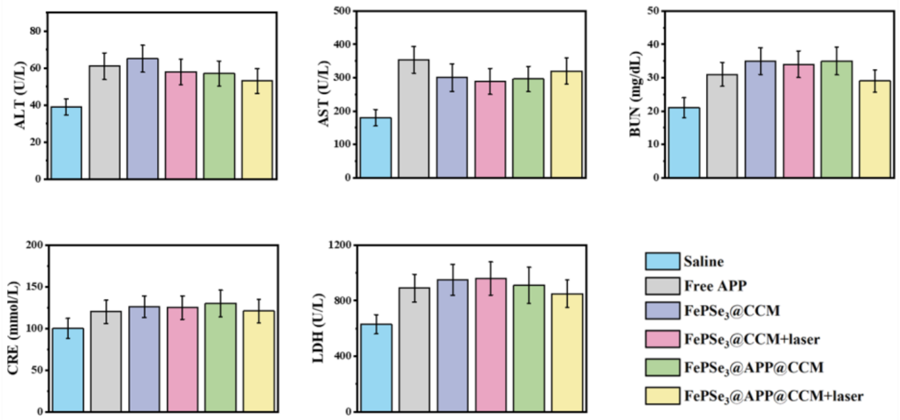

Figure S7. Serum biochemical indices (ALT, AST, BUN, CRE and LDH) of tumor-bearing mice after i.v. injection in different treatment groups for 25 days.

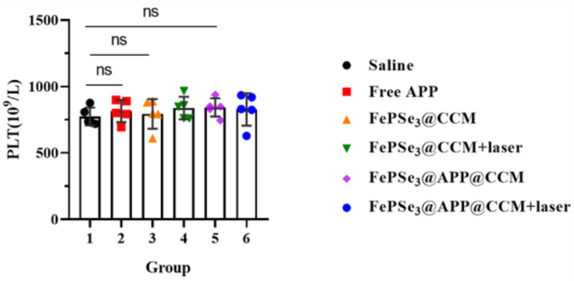

Figure S8. The peripheral platelet count of tumor-bearing mice in different treatment groups for 25 days. (n=5, mean±SD; ns=no significance)

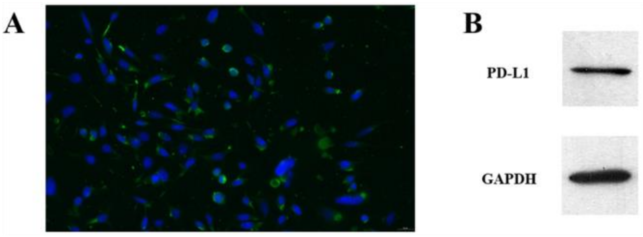

Figure S9. PD-L1 expression on CT26 cancer cells by immunofluorescence (A) and western blotting (B).

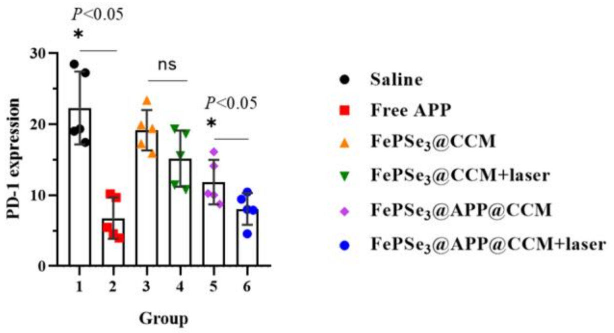

Figure S10. Statistical chart of PD-1 expression on PBMCs isolated from CT26 tumor-bearing mice in different treatment groups *in vivo*. (n=5, mean±SD; $*P<0.05$ and ns=no significance)

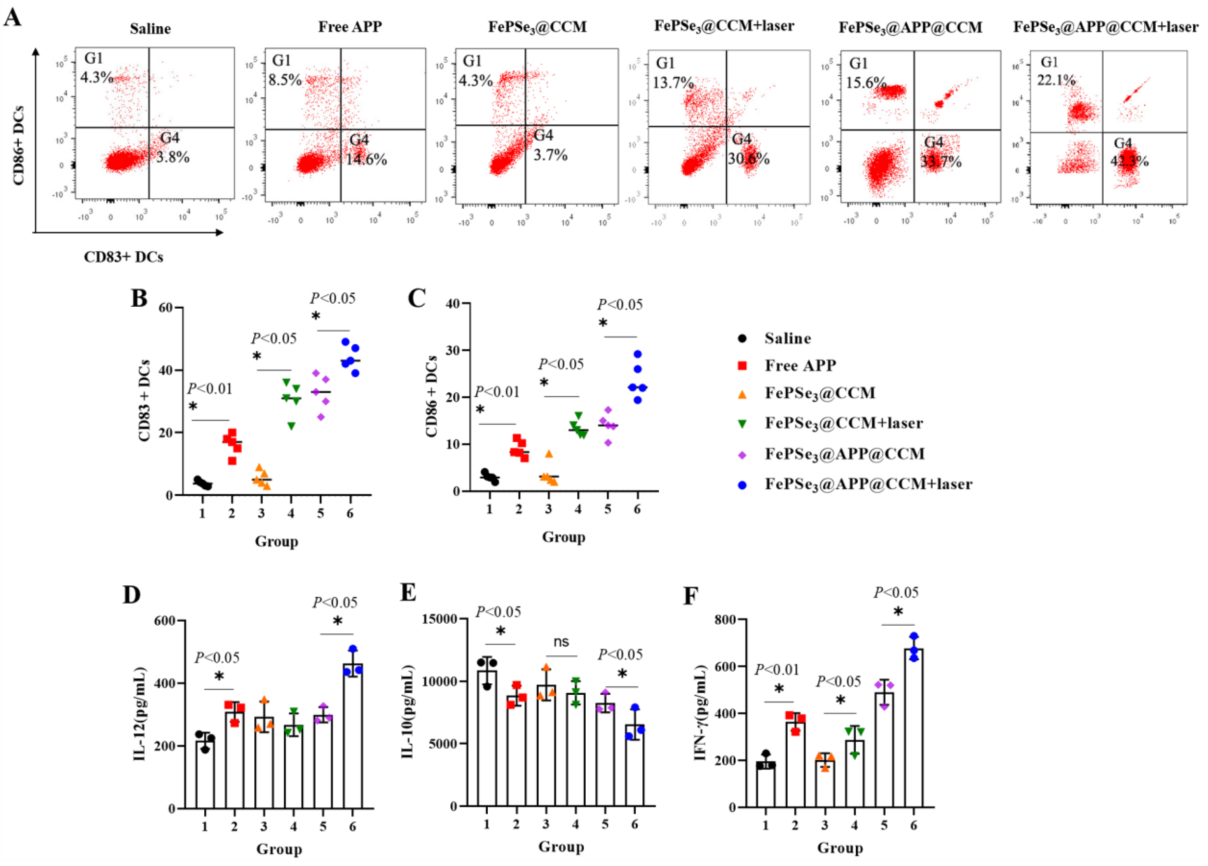

Figure S11. Immunotherapy effect of NSs on the CT26 tumor model. (A) Flow cytometry plots showing the mature DCs in CT26 tumor-bearing mouse spleens after different treatment

groups; (B and C) statistical chart of CD83+ and CD86+ expression on DCs extracted from the mouse spleens of different treatment groups (n=5, mean±SD; *P<0.05 and ns=no significance); (D-F) The expression quantity of cytokines including IL-12, IL-10 and IFN-γ detected in the blood serum from spleens of tumor-bearing mice in different treatment groups. (n=3, mean±SD; *P<0.05 and ns=no significance)

References:

1. Z. Y. Lei, Y. Y. Zhou and P. Y. Wu, *Small***2016**, 12, *3112*.
2. L. Z. He, T. Q. Nie, X. J. Xia, T. Liu, Y. Y. Huang, X. J. Wang and T. F. Chen, *Adv. Funct. Mater.***2019**, 29.
